# Advanced Glycation End Products Are Associated with Diabetes Status and Physical Functions in Patients with Cardiovascular Disease

**DOI:** 10.3390/nu14153032

**Published:** 2022-07-24

**Authors:** Tomoya Hirai, Kazuhiro Fujiyoshi, Satoru Yamada, Takuya Matsumoto, Junko Kikuchi, Kohki Ishida, Miwa Ishida, Minako Yamaoka-Tojo, Takayuki Inomata, Kyo Shigeta, Taiki Tojo

**Affiliations:** 1 Department of Cardiac Rehabilitation, Kitasato University Kitasato Institute Hospital, Minato-ku 108-8642, Japan; pt-hirai@insti.kitasato-u.ac.jp (T.H.); matsutaku0225@gmail.com (T.M.); junko@insti.kitasato-u.ac.jp (J.K.); shigetak@insti.kitasato-u.ac.jp (K.S.); 2 Department of Cardiovascular Medicine, Kitasato University School of Medicine, Sagamihara 252-0375, Japan; 3 Diabetes Center, Kitasato University Kitasato Institute Hospital, Minato-ku 108-8642, Japan; yamada-s@insti.kitasato-u.ac.jp; 4 Department of Cardiovascular Medicine, Kitasato University Kitasato Institute Hospital, Minato-ku 108-8642, Japan; ishikoh55@gmail.com (K.I.); miwai.1222@gmail.com (M.I.); ttojo@med.kitasato-u.ac.jp (T.T.); 5 Department of Rehabilitation, Kitasato University School of Allied Health Sciences, Sagamihara 252-0373, Japan; myamaoka@med.kitasato-u.ac.jp; 6 Department of Cardiovascular Medicine, Niigata University School of Medical and Dental Sciences, Niigata 951-8520, Japan; inotaka@med.niigata-u.ac.jp

**Keywords:** skin autofluorescence, glycemic control, muscle strength, exercise capacity

## Abstract

Advanced glycated end products (AGEs) accumulate systemically and cause diabetes complications. However, whether noninvasive measurable AGEs are associated with diabetes status and physical functions remains unclear. One hundred and ten patients with cardiovascular disease (CVD) who underwent outpatient cardiac rehabilitation were included. AGEs scores, using AGEs sensors, were evaluated concomitantly with a physical evaluation, including testing the isometric knee extension strength (IKES) and 6 min walking distance (6MWD). Thirty-three (30%) patients had a history of diabetes mellitus (DM). The AGEs score was not different in the presence of DM history (0.52 ± 0.09 vs. 0.51 ± 0.09, *p* = 0.768) and was not correlated with blood glucose (*r* = 0.001, *p* = 0.995). The AGEs score was positively correlated with hemoglobin A1c (HbA1c, *r* = 0.288, *p* = 0.004) and negatively correlated with physical functions (IKES, *r* = −0.243, *p* = 0.011; 6MWD, *r* = −0.298, *p* = 0.002). The multivariate analysis demonstrated that 6MWD was independently associated with a high AGEs score (>0.52). The AGEs score was associated with HbA1c, IKES, and 6MWD in patients with CVD. The AGEs score might be a useful indicator for evaluating not only glycemic control but also physical functions.

## 1. Introduction

Diabetes mellitus (DM) affects all-cause mortality in patients with cardiovascular disease (CVD) [1]. DM is an important factor in the secondary prevention and management of CVD. When hyperglycemia continues, serum advanced glycation end-products (AGEs) are produced during glucose metabolism [2]. Serum AGEs induce oxidative stress, which causes nitric oxide inactivation, inflammatory responses, thrombus formation, and progression of arteriosclerosis [3,4]. The accumulation of AGEs leads to the development of CVD. AGEs were evaluated by using a noninvasive physical method that uses the forearm and as a blood sampling test [5]. Skin autofluorescence (sAF) measured using the forearm was strongly correlated with AGEs from skin biopsy [6], although sAF did not match either serum or urine AGEs [7]. This sAF was associated with the duration of DM [8,9,10] and hemoglobin A1c [6,8,11]. Moreover, sAF was associated not only with DM but also with physical functions, including muscle strength and exercise capacity [11]. Furthermore, skin AGEs in patients undergoing cardiac rehabilitation are predictive of all-cause mortality and hospitalization for heart failure [12]. A meta-analysis reported that sAF levels measured by the forearm might be useful in assessing mortality risk in patients with CVD [13]. The most important aspect of CVD management is the noninvasive assessment of AGEs. Yamanaka et al. developed an AGEs sensor that can easily and quickly evaluate sAF by using a fingertip [14]. Several studies that used this new device showed that the AGEs score measured by the AGEs sensor was associated with serum AGEs [14] levels and glycation stress [15]. However, the relationship between AGEs score and clinical characteristics, including DM, has not been completely evaluated. Furthermore, the relationship between AGEs score and physical functions remains unclear. Thus, the purpose of this study was to (1) investigate the relationship between AGEs score and DM, (2) evaluate the relationship between AGEs score and glycemic control, and (3) assess the relationship between AGEs score and physical functions in patients with CVD.

## 2. Materials and Methods

### 2.1. Study Population

**Figure 1:**
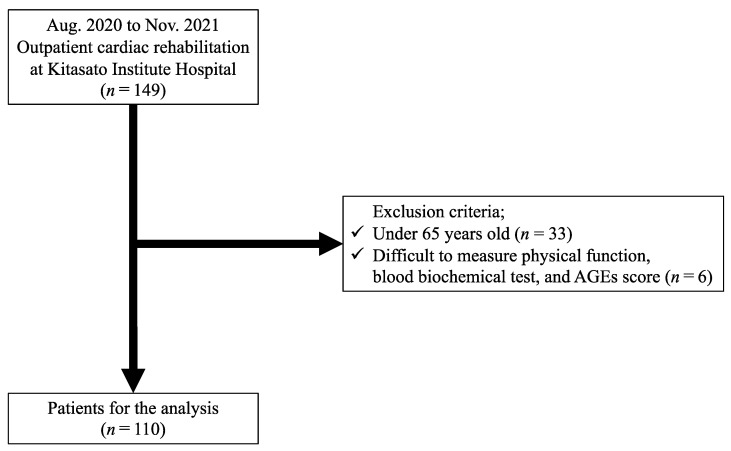
Study flowchart.

We conducted a single-center retrospective observational study between August 2020 and November 2021 at the cardiac rehabilitation center of Kitasato University Kitasato Institute Hospital. We enrolled 149 patients with CVD who underwent cardiac rehabilitation. CVD diagnosis included ischemic heart disease (myocardial infarction, angina pectoris, and vasospastic angina), heart failure, valvular heart disease, and atrial fibrillation. After excluding patients aged < 65 years (*n* = 33) and those who had difficulty measuring physical functions due to a decline in cognitive function or orthopedic disease (*n* = 6), 110 patients were finally included in the study (Figure 1). The Kitasato Institute Hospital Research Ethics Committee approved the study protocol (clinical trial registration number 21028).

### 2.2. Assessment of AGEs Score

To estimate the AGEs score, cardiac rehabilitation measurements of sAF levels were performed by using an AGEs sensor (SHARP, Kobe, Japan). AGEs have the property of emitting fluorescence when the specific excitation light irradiates them. The AGEs sensor irradiates the fingertips with excitation light, acquires percutaneous fluorescence of the fingertips, and measures skin autofluorescence [4]. The sAF levels were measured by using the middle finger of the left hand, in which the least amount of skin melanin was present [14]. We performed sAF measurements twice before cardiac rehabilitation and used the mean values for the analysis. The measured AGEs were expressed as the AGEs score in arbitrary units with an upper limit of 10.0 and a lower limit of 0.0. According to a recent manufacturer survey, 0.5 is an arbitrary unit that approximately corresponds to the average score of healthy Japanese patients aged 50 years. The AGEs sensor displayed a value when the coefficient of variation was less than 1%. A previous study has demonstrated that AGEs sensor is useful for the noninvasive assessment of glycation stress [15].

### 2.3. Assessment of Physical Functions

We evaluated handgrip strength (HGS), isometric knee extension strength (IKES), and 6 min walking distance (6MWD) as physical functions and used a dynamometer to measure HGS (TKK 5401; Takei, Tokyo, Japan). The patients performed two maximal isometric voluntary contractions of both hands for 3 s each while seated on a bench with the elbow flexed at 90°. The width of the dynamometer handle was adjusted for each patient to match their hand size. The highest strength measurement (kg) was used for the analysis. IKES was measured by using a handheld dynamometer to determine leg strength (μ-Tas; ANIMA, Tokyo, Japan). Briefly, with the patient seated in a chair with a non-extensible strap connecting the ankle to a strain gauge, 5 s of maximal isometric voluntary contractions of the quadriceps was collected twice for both legs, with the hip joint at approximately 90° flexion. Consecutive measurements were obtained for the right and left quadriceps muscles. The highest strength values on the right or left side were expressed as absolute values (kg) and relative to the body mass (%BM). The 6MWD was determined according to the guidelines of the American Thoracic Society, under the supervision of technicians. The patients were instructed to walk at their own pace along a straight, flat hallway from chair to chair, and the distance (in meters) was recorded after six minutes.

### 2.4. Definition

Hypertension was defined as an arterial blood pressure of ≥140/90 mmHg or the use of antihypertensive medication. Dyslipidemia (DL) was defined as low-density lipoprotein cholesterol ≥ 140 mg/dL, triglyceride ≥ 150 mg/dL, or the use of medication for DL. DM was defined as symptoms of diabetes plus random plasma glucose concentration ≥ 200 mg/dL, fasting plasma glucose concentration ≥ 126 mg/dL, or use of medication for DM. AGEs score, physical function, laboratory data, and clinical information were obtained within 2 weeks.

### 2.5. Statistical Analysis

**Figure 2:**
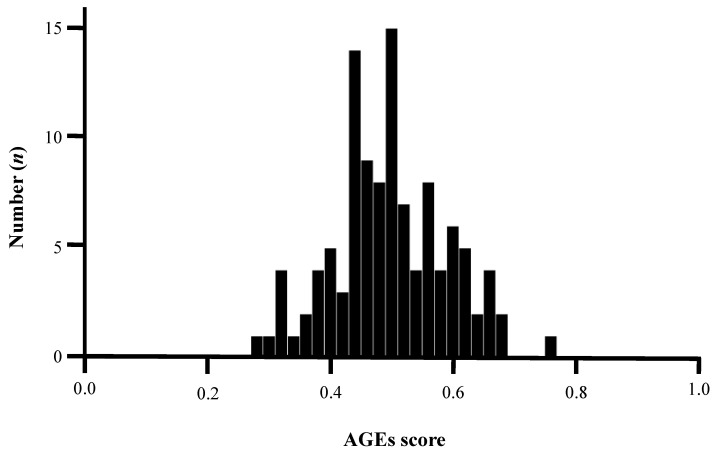
Normality test of AGEs score by Kolmogorov–Smirnov test. The average AGEs score was 0.51, and the median value was 0.52, indicating a normal distribution (*p* = 0.124). AGEs, advanced glycated end products.

Continuous variables with normal distribution were expressed as mean ± standard deviation (SD), whereas the median value with interquartile range was reported when the data were not normally distributed. The basic characteristics of patients with or without DM were compared. We analyzed the correlation between the AGEs score and patient characteristics. Continuous variables were analyzed by using a *t*-test. Categorical variables were reported as counts (%) and analyzed by using the chi-squared test. Multivariate regression analysis was performed to identify the factors associated with the presence of high AGEs score among variables with *p* < 0.050 in the univariate logistic regression analysis. Statistical significance was defined at *p* < 0.050. SPSS 27 version (IBM Corporation, Armonk, NY, USA) was used to perform all statistical analyses. Since related studies were limited, the cutoff value of the AGEs score is unknown. In this study, the AGEs score was normally distributed (Figure 2). We defined values above the median high as high AGEs score and below the median low as low AGEs score. Thus, the median AGEs score (0.52) was classified into two groups (high- and low-AGE patients) and subsequently analyzed.

## 3. Results

### 3.1. Clinical Characteristics between DM (+) and DM (−)

Among one hundred and ten older patients with cardiac rehabilitation, the number of patients with DM (+) and DM (−) were thirty-three (30%) and seventy-seven (70%), respectively. Table 1 describes the baseline clinical characteristics according to DM status. All clinical characteristics were similar between the two groups, except for male sex (79% vs. 58%, *p* = 0.041), DL (55% vs. 25%, *p* = 0.002), dipeptidyl peptidase-4 inhibitors (42% vs. 0%, *p* = 0.001), insulin (9% vs. 0%, *p* = 0.002), metformin (33% vs. 0%, *p* = 0.001), sodium-glucose cotransporter 2 inhibitors (61% vs. 12%, *p* = 0.001), blood glucose (139.6 ± 46.8 vs. 109.7 ± 22.4 mg/dL, *p* = 0.001), and HbA1c (7.0 ± 0.7 vs. 5.9 ± 0.5%, *p* = 0.001). There was no significant difference in the AGEs scores between DM (+) and DM (−) (0.52 ± 0.09 vs. 0.51 ± 0.09, *p* = 0.768).

### 3.2. Correlation between AGEs Score and Clinical Characteristics

**Figure 3:**
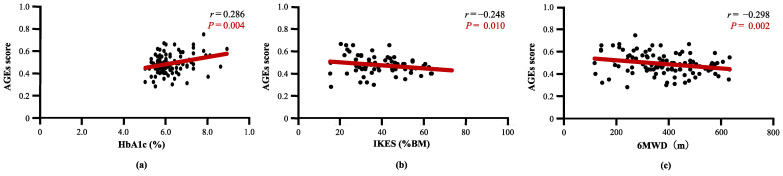
Correlation between AGEs score and clinical characteristics. (**a**) AGEs score was significantly correlated with HbA1c, (**b**) AGEs score was significantly correlated with Isometric knee extension strength, and (**c**) AGEs score was significantly correlated with 6MWD. AGEs, advanced glycated end products; HbA1c, hemoglobin-A1c; IKES, isometric knee extension strength; 6MWD, 6 min walking distance.

Table 2 describes the correlation between AGEs score and clinical characteristics. The AGEs score was not correlated with DM history (*r* = 0.038, *p* = 0.690), diabetic retinopathy (*r* = 0.133, *p* = 0.165), diabetic nephropathy (*r* = 0.109, *p* = 0.257), diabetic complications (*r* = 0.130, *p* = 0.175), or blood glucose (*r* = 0.001, *p* = 0.995). In the DM(−) population, there was no correlation between HbA1c and AGEs score (*r* = 0.299, *p* = 0.102). AGEs score was positively correlated with HbA1c (*r* = 0.286, *p* = 0.004) (Figure 3a) and negatively correlated with IKES (*r* = −0.248, *p* = 0.010) (Figure 3b) and 6MWD (*r* = −0.298, *p* = 0.002) (Figure 3c). The AGEs score was not correlated with other clinical characteristics in this study (Supplementary Figure S1).

### 3.3. Comparison of Clinical Characteristics between High and Low AGEs Score

The number of patients with a high and low AGEs score was fifty-three (48%) and fifty-seven (52%), respectively. Table 3 shows the comparison of clinical characteristics between the high AGE and low AGE score. All the clinical characteristics were similar between the two groups, except for HbA1c (6.4 ± 0.8 vs. 6.1 ± 0.7%, *p* = 0.044), HGS (21.1 ± 7.6 vs. 24.8 ± 8.8 kg, *p* = 0.023), IKES (36.5 ± 12.0 vs. 42.8 ± 13.5%BM, *p* = 0.013), and 6MWD (345 ± 132 vs. 410 ± 112 m, *p* = 0.010).

### 3.4. Physical Function and Presence of High AGEs Score

The univariate analysis showed that 6MWD (odds ratio (OR) 0.996; 95% confidence interval (CI) 0.993–0.999; *p* = 0.015) was significantly associated with the presence of a high AGEs score (>0.52) (Table 4). The multivariate analysis demonstrated that the 6MWD was independently associated with a high AGEs score (>0.52) (Table 5). Compared to the factors previously associated with AGEs [16,17,18], 6MWD was independently associated with a high AGEs score (Table 6 and Table 7).

## 4. Discussion

The main findings of this study were as follows: (1) there was no significant difference in AGEs score of patients with or without DM; (2) the AGEs score was significantly correlated with HbA1c but not with blood glucose; and (3) the AGEs score was also significantly correlated with physical functions, including IKES and 6MWD. In particular, the 6MWD was independently associated with a high AGEs score (>0.52).

### 4.1. AGEs Score and DM

In this study, AGEs score was not associated with a history of DM. Our results corroborated the findings of a previous study, which reported that AGEs measured by the forearm were not associated with a history of DM [12]. AGEs are metabolites of blood glucose and may not directly reflect the history of DM. AGEs include a variety of substances, such as pentosidine, carboxymethyl lysine, and pyrraline, which are produced by metabolites of blood glucose [19]. AGEs measured in the forearm have been shown to correlate with pentosidine levels [8]. AGEs measured at the fingertip were correlated with methylglyoxal 5-hydro-5-methylimidazolones [15]. Both are metabolites of blood glucose and may reflect glucose metabolism, which is not directly related to the history of DM. Furthermore, AGEs are not necessarily related to blood glucose because they are produced not only from glucose but also from fructose and aldehydes [20,21]. In contrast, it has also been reported that patients with DM have higher serum AGEs than those without DM [22]. These differences might be caused by variations in glucose metabolism, DM treatment, and subsequent DM status. Thus, the relationship between the AGEs score and a history of DM remains controversial. This small number of studies indicates that further data accumulation is needed in the future.

### 4.2. AGEs Score and HbA1c

The AGEs score was significantly correlated with HbA1c and was not correlated with blood glucose levels in this study. Previously, the serum AGEs were also strongly associated with HbA1c [22] but were not associated with blood glucose levels [11]. This is because AGEs are metabolized relatively slowly over weeks to months [23], reflecting the status of glycemic control over the medium-to-long-term from the vein to the skin [24]. Furthermore, HbA1c is an Amadori rearrangement substance produced by the same process as that of AGEs [25]. Therefore, AGEs are thought to reflect mid-to-long-term glycemic control rather than current glycemic control. Our study showed that the AGEs score may be useful as an indicator of noninvasive glycemic control in patients with CVD.

### 4.3. AGEs Score and Physical Functions

In this study, the AGEs score was also significantly correlated with physical functions, including IKES and 6MWD. The 6MWD was independently associated with a high AGEs score (>0.52). Previous studies have shown that forearm AGEs are significantly associated with reduced exercise tolerance [11] and physical functions, such as handgrip strength [26] and walking speed [27]. Accumulated AGEs increase muscle stiffness, reduce the viscoelastic properties of muscles, and impair muscle function [28]. In endothelial cells, AGEs affect endothelial dysfunction and loss of muscle mass and strength [29]. Clinically, serum AGEs are associated with evaluated endothelial function by brachial flow-mediated vasodilation [30]. Similarly, AGEs decrease exercise tolerance in the myocardium by inducing myocardial stiffness and diastolic dysfunction [3,31]. Therefore, patients with high AGEs have weaker HGS, IKES, and 6MWD than those with lower AGEs score. Furthermore, high physical function reduces the accumulation of AGEs. Since muscles consume glucose, AGEs accumulate less because of high muscle strength and exercise tolerance [32]. Several studies have clarified the relationship between physical activity and AGEs [33,34]. It has been reported that patients with higher physical activity have lower forearm AGEs [33]. Furthermore, AGEs are influenced by lifestyle habits such as physical activity, sleeping time, and cognitive function [35]. Individuals with low physical activity have been reported to accumulate more AGEs than those with high physical activity [34]. As mentioned above, the accumulation of AGEs affects muscle strength and exercise tolerance, whereas decreased physical activity accelerates the accumulation of AGEs. However, the causal relationship between AGEs and physical function remains unclear. A recent study suggests that long-term exercise may be reduced AGEs [36]. Further longitudinal studies are required to elucidate the causal relationship between AGEs accumulation and physical functions.

### 4.4. Limitations

This study had several limitations. First, this was a retrospective and observational study conducted at a single center with a limited number of patients. Second, there was no control group without CVD, because we focused on patients with CVD. Therefore, the results should be interpreted cautiously. Third, there was no complete removal of potential confounding factors that might affect the AGEs score (e.g., temperature, time, and season). Fourth, the AGEs score index may vary among the CVD types. Fifth, the results might have changed if a different cutoff AGEs score was applied. Sixth, there were no data on the diagnosis of DM in these patients. Seventh, this study did not consider the effects of dietary guidance or lifestyle. Eighth, three were no data of blood, plasma, or serum AGEs concentration. Thus, the interpretation needs caution about the association between the AGEs score and the presence of DM history. Ninth, this study included limited patients with chronic heart failure (52%), ischemia heart disease who underwent percutaneous coronary intervention (28%), valvular disease (6%), and atrial fibrillation (30%) who were outpatients for cardiac rehabilitation. The other cardiovascular disease could not speculate the AGEs score by sAF. Lastly, the findings of this observational study did not clarify the causal relationship between AGEs score and DM.

## 5. Conclusions

The AGEs score was associated with HbA1c level and physical functions in patients with CVD. The AGEs score might be a useful indicator for evaluating not only glycemic control but also physical functions.

## Figures and Tables

**Table 1:** Clinical characteristics of patients with and without DM.

	DM (+) (*n* = 33)	DM (−) (*n* = 77)	*p*-Value
Age, years	78.6 ± 7.2	78.6 ± 8.2	0.996
Male sex, *n* (%)	26 (79)	45 (58)	0.041 *
BMI, kg/m^2^	23.5 ± 3.7	22.4 ± 3.4	0.150
CHF			
HFrEF, *n* (%)	2 (6)	10 (13)	0.286
HFmrEF, *n* (%)	2 (6)	7 (9)	0.595
HFpEF, *n* (%)	12 (36)	25 (32)	0.692
PCI, *n* (%)	12 (36)	19 (25)	0.212
Valvular disease, *n* (%)	2 (6)	5 (6)	0.932
Atrial fibrillation, *n* (%)	6 (18)	27 (35)	0.077
Hypertension, *n* (%)	19 (58)	43 (56)	0.867
Hyperlipidemia, *n* (%)	18 (55)	19 (25)	0.002 *
Current smoker, *n* (%)	2 (6)	2 (3)	0.374
Medication			
DPP4i, *n* (%)	14 (42)	0 (0)	0.001 *
Insulin, *n* (%)	3 (9)	0 (0)	0.002 *
Metformin, *n* (%)	11 (33)	0 (0)	0.001 *
SGLT2i, *n* (%)	20 (61)	9 (12)	0.001 *
LVEF, %	58 ± 10	56 ± 12	0.563
LDL-C, mg/dL	79.8 ± 25.4	90.9 ± 31.5	0.092
HDL-C, mg/dL	58.5 ± 16.7	62.6 ± 14.6	0.262
TG, mg/dL	121.1 ± 71.4	116.9 ± 78.9	0.802
Blood glucose, mg/dL	139.6 ± 46.8	109.7 ± 22.4	0.001 *
HbA1c, %	7.0 ± 0.7	5.9 ± 0.5	0.001 *
Cr, mg/dL	1.2 ± 0.7	1.1 ± 0.4	0.363
eGFR, mL/min/1.73 m^2^	50.7 ± 17.5	51.4 ± 18.3	0.862
BNP, pg/dL	155.6 ± 189.1	261.3 ± 244.9	0.053
Handgrip strength, kg	24.7 ± 7.4	22.4 ± 8.8	0.196
IKES, %BW	42.6 ± 14.6	38.9 ± 12.5	0.179
6MWD, m	400 ± 129	374 ± 131	0.345
AGEs score	0.52 ± 0.09	0.51 ± 0.09	0.768

The date are means ± standard error or number (%). DM (+) vs. DM (−); * *p* < 0.050. AGEs, advanced glycated end products; BMI, Body Mass Index; BNP, brain natriuretic peptide; CHF, chronic heart failure; Cr, creatinine; DPP4i, dipeptidyl peptidase-4 inhibitors; eGFR, estimated glomerular filtration rate; HFmrEF, Heart Failure with mid-range Ejection Fraction; HFpEF, Heart Failure with preserved Ejection Fraction; HFrEF, Heart Failure with reduced Ejection Fraction; HbA1c, hemoglobin-A1c; HDL-C, HDL cholesterol; IKES, isometric knee extension strength; LDL-C, LDL cholesterol; LVEF, left ventricular ejection fraction; PCI, percutaneous coronary intervention; SGLT2i, sodium–glucose cotransporter 2 inhibitors; TG, triglyceride; 6MWD, 6 min walking distance.

**Table 2:** Correlation between AGEs score and clinical characteristics.

	*r*	*p*-Value		*r*	*p*-Value
Age	0.079	0.412	Current smoker	−0.082	0.396
Male	−0.077	0.424	LVEF	−0.019	0.413
BMI	0.110	0.275	LDL-C	−0.038	0.708
HFrEF	0.041	0.669	HDL-C	−0.025	0.817
HFmrEF	0.028	0.772	TG	0.030	0.771
HFpEF	0.048	0.620	Blood glucose	0.001	0.995
PCI	−0.012	0.899	HbA1c	0.288	0.004 *
Valvular disease	0.066	0.496	Cr	0.178	0.062
Atrial fibrillation	−0.058	0.546	eGFR	−0.184	0.054
Hypertension	0.081	0.401	Diabetic nephropathy	0.109	0.257
Hyperlipidemia	−0.083	0.386	BNP	−0.065	0.526
Diabetes mellitus	0.038	0.690	Hand grip strength	−0.127	0.187
Diabetic retinopathy	0.133	0.165	IKES	−0.243	0.011 *
Diabetic complications	0.130	0.175	6MWD	−0.298	0.002 *

Note: *r* indicates the correlation coefficient; * *p* < 0.050. AGEs, advanced glycated end products; BMI, Body Mass Index; BNP, brain natriuretic peptide; Cr, creatinine; eGFR, estimated glomerular filtration rate; HFmrEF, Heart Failure with mid-range Ejection Fraction; HFpEF, Heart Failure with preserved Ejection Fraction; HFrEF, Heart Failure with reduced Ejection Fraction; HbA1c, hemoglobin-A1c; HDL-C, HDL cholesterol; IKES, isometric knee extension strength; LDL-C, LDL cholesterol; LVEF, left ventricular ejection fraction; PCI, percutaneous coronary intervention; TG, triglyceride; 6MWD, 6 min walking distance.

**Table 3:** Comparison of baseline characteristics of patents with high AGEs and low AGEs score.

	High AGEs Score (*n* = 53)	Low AGEs Score (*n* = 57)	*p*-Value
Age, years	79.6 ± 7.7	77.8 ± 8.0	0.217
Male sex, *n* (%)	28 (56)	43 (72)	0.087
BMI, kg/m^2^	23.3 ± 3.5	22.2 ± 3.5	0.113
CHF			
HFrEF, *n*(%)	5 (10)	7 (12)	0.780
HFmrEF, *n*(%)	6 (12)	3 (5)	0.182
HFpEF, *n*(%)	19 (38)	18 (30)	0.377
PCI, *n* (%)	13 (26)	18 (30)	0.642
Valvular disease, *n* (%)	4 (8)	3 (5)	0.521
Atrial fibrillation, *n* (%)	12 (24)	21 (35)	0.210
Hypertension, *n* (%)	33 (66)	29 (48)	0.063
Hyperlipidemia, *n* (%)	16 (32)	21 (35)	0.740
Diabetes mellitus, *n* (%)	15 (30)	18 (30)	1.000
Diabetic retinopathy, *n* (%)	3 (6)	2 (4)	0.588
Diabetic complications, *n* (%)	4 (8)	3 (5)	0.624
Current smoker, *n* (%)	0 (0)	4 (7)	0.063
Medication			
DPP4i, *n* (%)	9 (17)	5 (9)	0.197
Insulin, *n* (%)	0 (0)	3 (5)	0.090
Metformin, *n* (%)	6 (11)	5 (9)	0.656
SGLT2i, *n* (%)	17 (32)	12 (21)	0.190
LVEF, %	56.4 ± 10.6	56.7 ± 12.4	0.916
LDL-C, mg/dL	83.6 ± 30.8	90.5 ± 29.5	0.259
HDL-C, mg/dL	61.0 ± 14.0	61.9 ± 16.4	0.782
TG, mg/dL	115.1 ± 67.5	120.6 ± 83	0.725
Blood glucose, mg/dL	118.7 ± 38.6	119.1 ± 31.2	0.952
HbA1c, %	6.4 ± 0.8	6.1 ± 0.7	0.044 *
Cr, mg/dL	1.2 ± 0.7	1.1 ± 0.4	0.238
eGFR, mL/min/1.73 m^2^	48.8 ± 20.5	53.2 ± 15.5	0.195
Diabetic nephropathy, *n* (%)	2 (4)	1 (2)	0.516
BNP, pg/dL	230 ± 211	236 ± 255	0.908
Handgrip strength, kg	21.1 ± 7.6	24.8 ± 8.8	0.023 *
IKES, %BW	36.5 ± 12.0	42.8 ± 13.5	0.013 *
6MWD, m	345 ± 132	410 ± 112	0.010 *
AGEs score	0.57 ± 0.06	0.43 ± 0.06	<0.001 *

The date are means ± standard error or number (%). High AGEs score vs. low AGEs score; * *p* < 0.050. AGEs, advanced glycated end products; BMI, Body Mass Index; BNP, brain natriuretic peptide; CHF, Chronic heart failure; Cr, creatinine; DPP4i, dipeptidyl peptidase-4 inhibitors; eGFR, estimated glomerular filtration rate; HFmrEF, Heart Failure with mid-range Ejection Fraction; HFpEF, Heart Failure with preserved Ejection Fraction; HFrEF, Heart Failure with reduced Ejection Fraction; HbA1c, hemoglobin-A1c; HDL-C, HDL cholesterol; IKES, isometric knee extension strength; LDL-C, LDL cholesterol; LVEF, left ventricular ejection fraction; PCI, percutaneous coronary intervention; SGLT2i, sodium–glucose cotransporter 2 inhibitors; TG, triglyceride; 6MWD, 6 min walking distance.

**Table 4:** Univariate analysis for the presence of high AGEs score (>0.52).

Variable	Univariate Analysis		
	OR	95% CI	*p*-Value
Age, per year	1.036	0.986–1.087	0.160
Male sex	1.422	0.649–3.115	0.379
BMI, per kg/m^2^	1.078	0.963–1.208	0.193
HFrEF	0.922	0.278–3.057	0.894
HfmrEF	0.725	0.184–2.856	0.645
HfpEF	0.595	0.268–1.321	0.202
PCI	1.420	0.614–3.285	0.412
Valvular disease	0.349	0.065–1.882	0.221
Atrial fibrillation	0.983	0.436–2.223	0.967
Hypertension	0.540	0.251–1.159	0.114
Hyperlipidemia	1.349	0.609–2.990	0.461
Diabetes mellitus	0.826	0.365–1.870	0.647
Diabetic retinopathy	0.606	0.097–3.777	0.592
Diabetic complications	0.681	0.145–3.194	0.626
Current smoker	2.081	0.951–4.557	0.067
LVEF, per %	0.995	0.952–1.030	0.785
Hb, per g/dL	0.889	0.723–1.095	0.268
LDL-C, per mg/dL	0.999	0.985–1.013	0.925
HDL-C, per mg/dL	0.997	0.970–1.025	0.844
TG, per mg/dL	1.000	0.995–1.005	0.906
Blood glucose, per mg/dL	1.003	0.991–1.016	0.583
HbA1c, per %	1.506	0.866–2.558	0.130
Cr, per mg/dL	1.371	0.667–2.820	0.391
eGFR, per mL/min/1.73 m^2^	0.988	0.968–1.009	0.276
Diabetic nephropathy	0.455	0.040–5.174	0.525
BNP, per pg/dL	0.999	0.998–1.001	0.518
Handgrip strength, per kg	0.963	0.921–1.007	0.101
IKES, per %BW	0.973	0.945–1.001	0.059
6MWD, per m	0.996	0.993–0.999	0.015 *

OR, odds ratio; CI, confidence interval; * *p* < 0.050. AGEs, advanced glycated end products; BMI, Body Mass Index; BNP, brain natriuretic peptide; Cr, creatinine; eGFR, estimated glomerular filtration rate; HfmrEF, Heart Failure with mid-range Ejection Fraction; HfpEF, Heart Failure with preserved Ejection Fraction; HfrEF, Heart Failure with reduced Ejection Fraction; HbA1c, hemoglobin-A1c; HDL-C, HDL cholesterol; IKES, isometric knee extension strength; LDL-C, LDL cholesterol; LVEF, left ventricular ejection fraction; PCI, percutaneous coronary intervention; TG, triglyceride; 6MWD, 6 min walking distance.

**Table 5:** Multivariate analysis for high AGEs score (>0.52).

Variable	Multivariate Analysis			Multivariate Analysis		
	OR	95% CI	*p*-Value	OR	95% CI	*p*-Value
6MWD, per m	0.996	0.993–0.999	0.012 *	0.997	0.994–0.999	0.048 *
Diabetes mellitus	0.645	0.273–1.524	0.317			
Blood glucose, per mg/dL				1.005	0.992–1.017	0.451

OR, odds ratio; CI, confidence interval; * *p* < 0.050. AGEs, advanced glycated end products; 6MWD, 6 min walking distance.

**Table 6:** Multivariate analysis for high AGEs score (>0.52).

Variable	Multivariate Analysis			Multivariate Analysis		
	OR	95% CI	*p*-Value	OR	95% CI	*p*-Value
6MWD, per m	0.996	0.993–1.000	0.035 *	0.996	0.992–0.999	0.023 *
HbA1c, per %	1.646	0.946–2.864	0.078			
Age, per year				0.989	0.932–1.050	0.720

OR, odds ratio; CI, confidence interval; * *p* < 0.050. AGEs, advanced glycated end products; HbA1c, hemoglobin-A1c; 6MWD, 6 min walking distance.

**Table 7:** Multivariate analysis for high AGEs score (>0.52).

Variable	Multivariate Analysis			Multivariate Analysis			Multivariate Analysis		
	OR	95% CI	*p*-Value	OR	95% CI	*p*-Value	OR	95% CI	*p*-Value
6MWD, per m	0.995	0.992–0.999	0.007 *	0.996	0.993–1.000	0.025 ^*^	0.997	0.993–1.000	0.045 *
BMI, per kg/m^2^	1.102	0.975–1.245	0.121						
Current smoker				1.613	0.712–3.655	0.252			
eGFR, per mL/min/1.73 m^2^							0.990	0.966–1.015	0.431

OR, odds ratio; CI, confidence interval; * *p* < 0.050. AGEs, advanced glycated end products; BMI, Body Mass Index; eGFR, estimated glomerular filtration rate; 6MWD, 6 min walking distance.

## Data Availability

Not applicable.

## Supplementary Materials

The following supporting information can be downloaded at: https://www.mdpi.com/article/10.3390/nu14153032/s1, Figure S1: Correlation between AGEs score and clinical characteristics.
