# Supplementary material for: Advanced Glycation End Products Are Associated with Diabetes Status and Physical Functions in Patients with Cardiovascular Disease
Source: Nutrients. 2022 Jul 24;14(15):3032. doi: 10.3390/nu14153032 (PMC9330730; doi:10.3390/nu14153032)
Supplement: Supplementary file 1 [file nutrients-14-03032-s001.zip › nutrients-1828004-supplementary.pdf]

Supplemental material

Supplementary Figure S1. Correlation between AGEs score and clinical characteristics.

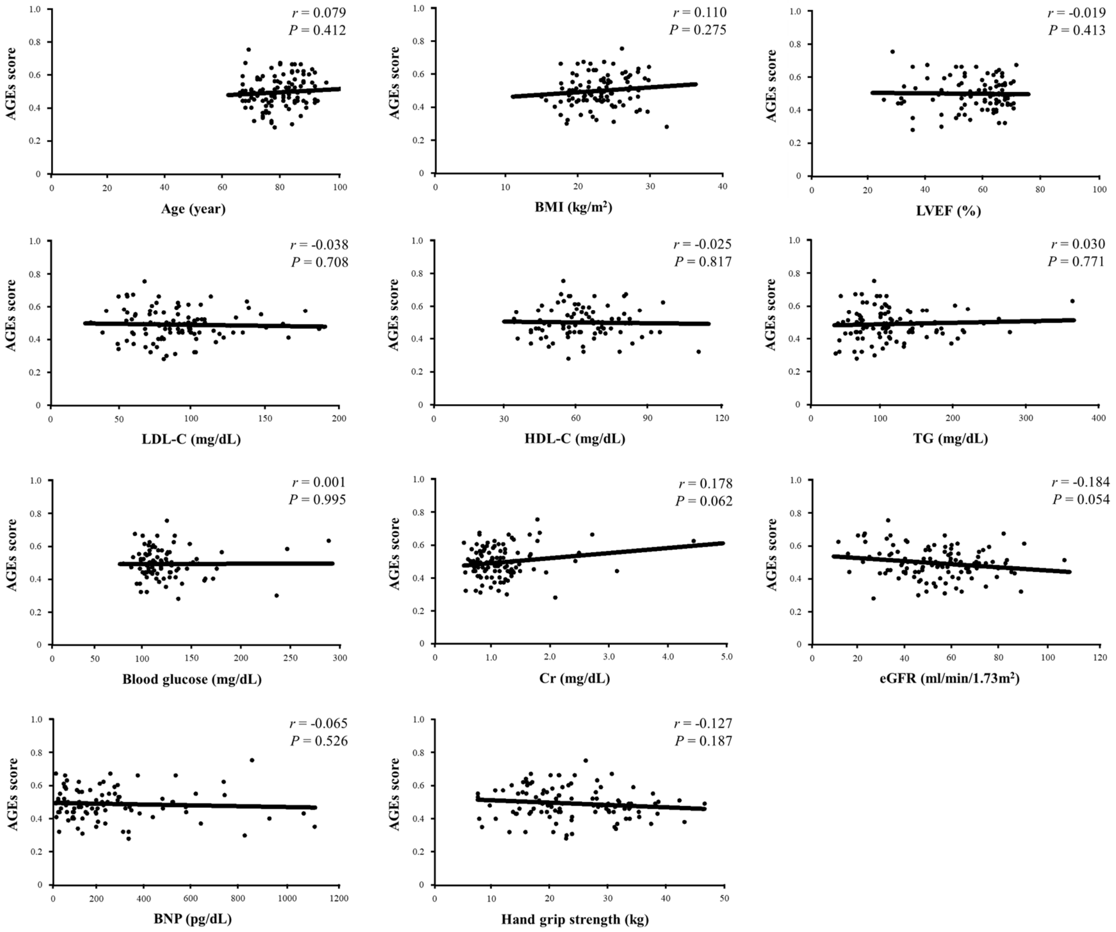

r indicates the correlation coefficient. BMI, Body Mass Index; BNP, brain natriuretic peptide; Cr, creatinine; eGFR, estimated glomerular filtration rate; HDL-C, HDL-cholesterol; LDL-C, LDL-cholesterol; LVEF, left ventricular ejection fraction; TG, triglyceride.
